# The P2X7 ion channel is dispensable for energy and metabolic homeostasis of white and brown adipose tissues

**DOI:** 10.1007/s11302-020-09738-7

**Published:** 2020-10-06

**Authors:** Tian Tian, Markus Heine, Ioannis Evangelakos, Michelle Y. Jaeckstein, Nicola Schaltenberg, Tobias Stähler, Friedrich Koch-Nolte, Manju Kumari, Joerg Heeren

**Affiliations:** 1 Department of Biochemistry and Molecular Cell Biology, University Medical Center Hamburg-Eppendorf, 20246 Hamburg, Germany; 2 Institute of Immunology, University Medical Center Hamburg-Eppendorf, 20246 Hamburg, Germany

**Keywords:** Purinergic signaling, Adipose tissue, P2X7 ion channel, Energy metabolism, Obesity

## Abstract

Several studies suggest a role of extracellular adenine nucleotides in regulating adipose tissue functions via the purinergic signaling network. Metabolic studies in mice with global deletion of the purinergic receptor P2X7 on the C57BL/6 background indicate that this receptor has only a minor role in adipose tissue for diet-induced inflammation or cold-triggered thermogenesis. However, recent data show that a polymorphism (P451L) present in C57BL/6 mice attenuates P2X7 receptor function, whereas BALB/c mice express the fully functional P451 allele. To determine the potential role of *P2rx7* under metabolic and thermogenic stress conditions, we performed comparative studies using male *P2rx7* knockout (KO) and respective wild-type controls on both BALB/c and C57BL/6 backgrounds. Our data show that adipose *P2rx7* mRNA levels are increased in obese mice. Moreover, *P2rx7* deficiency results in reduced levels of circulating CCL2 and IL6 with a moderate effect on gene expression of pro-inflammatory markers in white adipose tissue and liver of BALB/c and C57BL/6 mice. However, P2X7 expression does not alter body weight, insulin resistance, and hyperglycemia associated with high-fat diet feeding on both genetic backgrounds. Furthermore, deficiency of *P2rx7* is dispensable for energy expenditure at thermoneutral and acute cold exposure conditions. In summary, these data show that—apart from a moderate effect on inflammatory cytokines—P2X7 plays only a minor role in inflammatory and thermogenic effects of white and brown adipose tissue even on the BALB/c background.

**Electronic supplementary material:**

The online version of this article (10.1007/s11302-020-09738-7) contains supplementary material, which is available to authorized users.

## Introduction

Adipose tissue is a complex organ with an important role in endocrine, metabolic, and immune regulatory processes. In mammals, there are two types of adipose tissues: white adipose tissue (WAT) composed of white adipocytes that stores energy in the form of triglycerides and brown adipose tissue (BAT), characterized by uncoupling protein 1 (UCP1) expressing brown adipocytes which combusts energy-rich nutrients for heat production [1–3]. The balanced function of the two types of adipose tissues plays a major role in maintaining whole body energy homeostasis. Accordingly, adipose tissue dysfunction is closely related to metabolic disorders such as obesity, insulin resistance, and hyperglycemia [4, 5]. It has been established that chronic, subclinical inflammation in WAT depots is a key player in the development of insulin resistance in obesity [6]. Strong infiltration of immune cells, especially macrophages, into WAT takes place upon exposure to an obesogenic high-fat diet which is associated with increased production of cytokines and chemokines [7, 8]. This is correlated with local expression of genes for inflammatory mediators such as *Tnf*, *Il6*, and *Ccl2* [9]. Adipose tissue mRNA as well as circulating CCL2 and IL6 levels correlate positively with the degree of corpulence and insulin resistance in obese mice and humans [9–12].

Recent studies have indicated that extracellular adenosine 5-triphosphate (ATP) is involved in the regulation of tissue inflammation via a purinergic signaling network, which involves the release of ATP, ectoenzymes hydrolyzing ATP to adenosine such as CD39 (ectonucleoside triphosphate diphosphohydrolase-1) and CD73 (ecto-5′-nucleotidase) [13, 14]. Importantly, extracellular ATP activates purinergic receptors of the P2X and P2Y families. Unlike the G protein–coupled P2Y receptors, P2X receptors are ligand-gated ion channels. Of the 7 members of P2X receptor family, P2X7 has been studied intensively due to its unique ability to activate the inflammasome [15]. Increased levels of extracellular ATP activate P2X7, leading to rapid K^+^ efflux, activation of the caspase-1 containing inflammasome NLRP3, and pore formation by pannexins and gasdermin D [16]. The activation of P2X7 is also associated with other cell-specific signaling pathways involved in inflammation, such as inflammatory cytokine production, reactive oxygen and nitrogen species formation, and protease activation [17–19].

The murine *P2rx7* gene has a functional single nucleotide polymorphism (SNP) at position 451 named as P451L [20]. Compared with the wild-type allele 451P, the 451L allele is reported to have lower sensitivity to ATP in T cells [20]. Further studies indicate that P451L SNP is associated with *P2rx7* induced functions including pore formation, cell death, and intracellular Ca^2+^ waves [21, 22]. The *P2rx7* alleles P451 and 451L are differently distributed in mouse strains, whereas BALB/c mice encode the full-functional P451 allele while C57BL/6 mice carry the 451L allele [20, 23, 24].

The aim of this study was to determine the expression of purinergic receptors (*P2rx4*, *P2rx5, P2rx7*) in adipose tissue of obese mice and in isolated adipocytes, endothelial cells, and tissue-resident macrophages of adipose tissue under normal and cold adaptation housing condition. Moreover, we investigated the role of P2X7 in diet-induced obesity, adipose tissue inflammation, and energy expenditure on BALB/c and C57BL/6 genetic background. The results of our metabolic studies show that despite reduced systemic inflammation, *P2rx7* deficiency does not alter diet-induced obesity, insulin resistance, and hyperglycemia associated with high-fat diet (HFD) feeding or thermogenic responses in BALB/c and C57BL/6 backgrounds.

## Materials and methods

### Experimental mice and diet study

*P2rx7* KO mice [25] were backcrossed to C57BL/6 and BALB/c mice for 16 generations and were maintained under specific pathogen-free conditions at the central animal facility of the UKE. All experiments were performed according to state guidelines with approval of the local institutional regulatory committee. We used age-matched male BALB/c and C57BL/6J wild-type mice and *P2rx7* KO mice. Mice were maintained on a standard chow diet (19.10% protein, 4% fat, 6% fiber, from Altromin Spezialfutter GmbH&Co, Germany) under a regular 12-h light/12-h dark cycle at 22 °C temperature. High-fat diet experiments in BALB/c and C57BL/6J wild-type and *P2rx7* KO mice were performed by feeding 6-week-old male mice a high-fat diet (20% protein, 35.6% fat, 0.3% fiber, 23.2% sugar, from ssniff Spezialitäten GmbH, Germany) for 16 weeks in a humidity-controlled climate chamber at thermoneutral (30 °C) or room temperature (22 °C) conditions. Body weight was measured weekly. Whole body composition was measured by echoMRI (EchoMRI™, USA). At the end of experiments, mice were sacrificed by cervical dislocation, and harvested tissues were either formalin-fixed for histology analyses or snap frozen in liquid nitrogen for quantitative PCR and Western blot analyses. Frozen tissues were stored at − 80 °C.

### Energy expenditure measurement

Metabolic rate was measured by indirect calorimetry in metabolic cages (TSE system GmbH, Germany) as described [26]. The system was operated according to the manufactures guidelines. All mice were acclimatized to monitoring cages for 48 h prior to the beginning of physiological parameters recording. In this climate-controlled indirect calorimetry setup, O_2_ consumption, CO_2_ production, food and drink intake, as well as activity were monitored. Mice were housed under 12 to 12-h light/dark cycle. In order to determine thermogenic capacity in *P2rx7* KO and wild-type mice, the temperature was gradually decreased by 4–5 °C (from 3 to 6 °C) at 7 a.m. each day.

### Glucose tolerance test

To determine glucose tolerance, mice were fasted for 6 h followed by an intraperitoneal injection of glucose (Sigma) at 1 g/kg body weight. Blood samples were collected at 0, 15, 30, 60, 90, and 120 min. Glucose levels were measured using Accuchek Aviva (Roche).

### Plasma and liver parameters

Plasma and liver cholesterol and triglyceride levels were quantified using commercial kits (Roche) that were adapted to 96-well microtiter plates according to manufacturer’s instructions. Ultra-Sensitive Mouse Insulin ELISA kit (Crystal Chem) was used for the quantitative determination of insulin in mouse plasma.

### Tissue histology

Adipose tissues were fixed in 3.7% formalin for 24 h, embedded in paraffin, and sectioned by the Leica RM 2245 microtome for hematoxylin and eosin (H&E) staining. For immunohistochemistry, sections were subjected to antigen retrieval in citrate buffer (pH 6). After blocking with 3% BSA for 1 h at room temperature, primary rat monoclonal anti-MAC-2 antibody was diluted 1:250 in 3% BSA and incubated over night at 4 °C. HRP-anti-rat secondary antibody was diluted 1:250 and incubated for 1 h at room temperature. High resolution digital images were taken using the Nikon eclipse Ti Microscope.

### Isolation of adipocytes, macrophages, and endothelial cells from adipose tissue

After collagenase (Sigma, USA) digestion of WAT and BAT harvested from 4 mice, large adipocytes were separated by low speed centrifugation. Next, magnetic separation was performed according to the manufacturer’s instructions using an autoMACS pro Separator (Miltenyi Biotec GmbH, Germany). The cell pellet was re-suspended in 900 μl MACS sorting buffer and incubated with 100 μl Cd11b MicroBeads (MiltenyiBiotec GmbH, Germany) per 10^7^ total cells at 4 °C for 15 min. After centrifugation, the cell pellet was re-suspended and cell suspension was applied on LS columns (MiltenyiBiotec GmbH, Germany). The magnetically labeled Cd11b+ macrophage cell fraction was collected from LS columns. The flow through fraction was incubated with Cd31 MicroBeads (MiltenyiBiotec GmbH, Germany) and applied on LS columns to collect Cd31+ endothelial cells. The last flow through fraction was obtained as adipocytes.

### RNA isolation and qRT-PCR

qPCR analysis was performed as described previously [27]. Briefly, RNA was extracted using the Nucleo Spin RNA® (Macherey-Nagel™, Germany) according to the manufacturer’s protocol. RNA was quantified using NanoDrop (Thermo Scientific, Wilmington, DE) and converted to cDNA using SuperScript III Reverse Transcriptase (Invitrogen). Real-time qPCR was performed on a 7900HT Sequence Detection System (Applied Biosystems, USA) using TaqMan Assay-on-Demand primer sets (Applied Biosystems) (*Tbp*:Mm00446973_m, *Cd39*:Mm00515447_m1, *Cd73*:Mm00501910_m1, *P2rx4*:Mm00501787_m1, *P2rx7*:Mm01199500_m1, *P2rx5*:Mm00473677_m1, *Adipoq*:Mm00456425_m1, *Gpihbp1*:Mm01205849_g1, *Emr1*:Mm00802530_m1, *Ucp1*:Mm00494069_m1, *Arg1*:Mm00475988_m1, *Tnf*:Mm00443258_m1, *Il1b*:Mm00434228_m1, *Il6*:Mm00446190_m1, *Ccl2*:Mm00441242_m1, *Nlrp3*:Mm00840904_m1, *Cd4*:Mm00442754_m1, *Cd8b1*:Mm00438116_m1, *Acta2*: Mm01546133_m1, *Col1a1:*Mm00801666_g1, *Timp1*:Mm00441818_m1, *Tgfb1*: Mm00441724_m1, *Mmp12*:Mm00500554_m1, *Mmp13*:Mm00439491_m1, *Trem2*:Mm00451744_m1). Cycling parameters were as follows: 1 cycle of 95 °C for 10 m, 40 cycles of 95 °C for 15 s then 60 °C for 60 s, followed by melt curve analysis. Cycle thresholds (Ct values) were normalized to those of the *Tbp* housekeeping gene.

### Protein extraction and Western blotting

Total lysates were prepared by homogenizing various tissues in RIPA buffer supplemented with protease inhibitors (Roche) and phosphatase inhibitors (Sigma). Protein samples (25 μg per lane) were separated by SDS-PAGE. Blots were blocked for 2 h in PanReac Blocking buffer (AppliChem), incubated for 1 h with appropriate primary antibody and 2 h with HRP-conjugated secondary antibody. Polyclonal rabbit antibodies directed against the C-terminal peptides of P2X4 and P2X7 were from Abcam (Cat. No. ab243734, Cat. No. ab229453). The P2X7 antibody detected two bands at around 75 kDa in BAT of Balb/c mice. The upper band was also detectable in knockout mice, indicating that as a non-specific cross-reactivity of the antibody. P2X5 rabbit polyclonal antibody was purchased from Thermo Fisher (Cat. No. PA5-41079) and the loading control γ-tubulin rabbit monoclonal antibody from Abcam (Cat. No. ab179503). The secondary antibody, goat-anti-rabbit IgG horseradish peroxidase (HRP), was purchased from Bio-Rad. Detection was performed on Amersham Imager600 using luminol and para-hydroxycoumarinic acid–based chemiluminescence substrate.

### Statistical analysis

Results are expressed as mean ± SEM. Comparisons between groups were made using either unpaired, two-tailed Student’s *t* test of the SPSS software for two-group comparisons or the two-way ANOVA test with Bonferroni post-tests of the PRISM software for multigroup comparisons. *P* < 0.05 was considered statistically significant.

## Results

### Gene expression analysis of P2X receptors and nucleotide-metabolizing enzymes in WAT and BAT of lean, DIO, and *ob*/*ob* mice

**Fig. 1:**
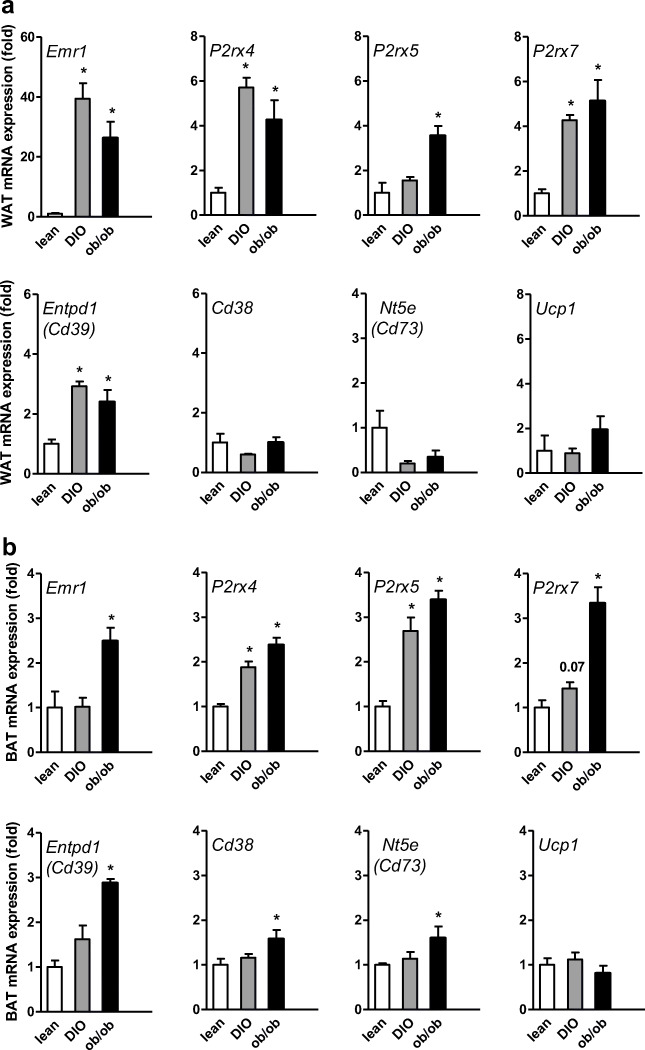
Gene expression analysis in **a** WAT and **b** BAT of lean, DIO, and *ob*/*ob* mice. C57BL6/J wild-type (WT) mice were fed a normal chow (lean) or a diabetogenic high-fat diet for 16 weeks to generate lean or diet-induced obese (DIO) mice. From these mice as well as from leptin-deficient *ob*/*ob* mice, the expression of genes important for extracellular adrenergic signaling was determined by qPCR. Data are presented as mean ± SEM, *P < 0.05

To assess the effect of nutritional state on expression of enzymes and receptors involved in purinergic signaling, we performed qPCR analyses of WAT and BAT isolated from lean and obese mice. Obesity is well-known to result in infiltration of pro-inflammatory macrophages [8]. In line, we detected increased expression of *Emr1* in WAT of both dietary (diet-induced obesity: DIO) and genetic (*ob*/*ob*) models of obesity (Fig. 1a). This inflammatory response in WAT of these mice was associated with the increased gene expression of the purinergic receptors *P2rx4*, *P2rx5*, and *P2rx7* as well as marked changes in gene expression levels of nucleotide-metabolizing enzymes CD39 (encoded by *Entpd1*) but not *Cd38,* CD73 (encoded by *Nt5e*), and *Ucp1* (Fig. 1a). Next to WAT, obesity and high fat diet feeding have also pronounced effects on BAT inflammatory status and results in a WAT-like morphology which is referred to as BAT whitening [2, 28]. In BAT of *ob*/*ob* mice, increased *Emr1* expression was observed (Fig. 1b). In whitened BAT of DIO and *ob*/*ob* mice, we also detected increased gene expression of *P2rx4*, *P2rx5*, and *P2rx7* (Fig. 1b). Similar to *Emr1*, higher levels of *Entpd1*, *Cd38*, and *Nt5e* were found in BAT of *ob*/*ob* mice, while no obesity-associated changes in *Ucp1* expression were detected. Overall, these data suggest that purinergic P2X receptors could be involved in the regulation of obesity-associated inflammatory responses in WAT and BAT.

**Fig. 2:**
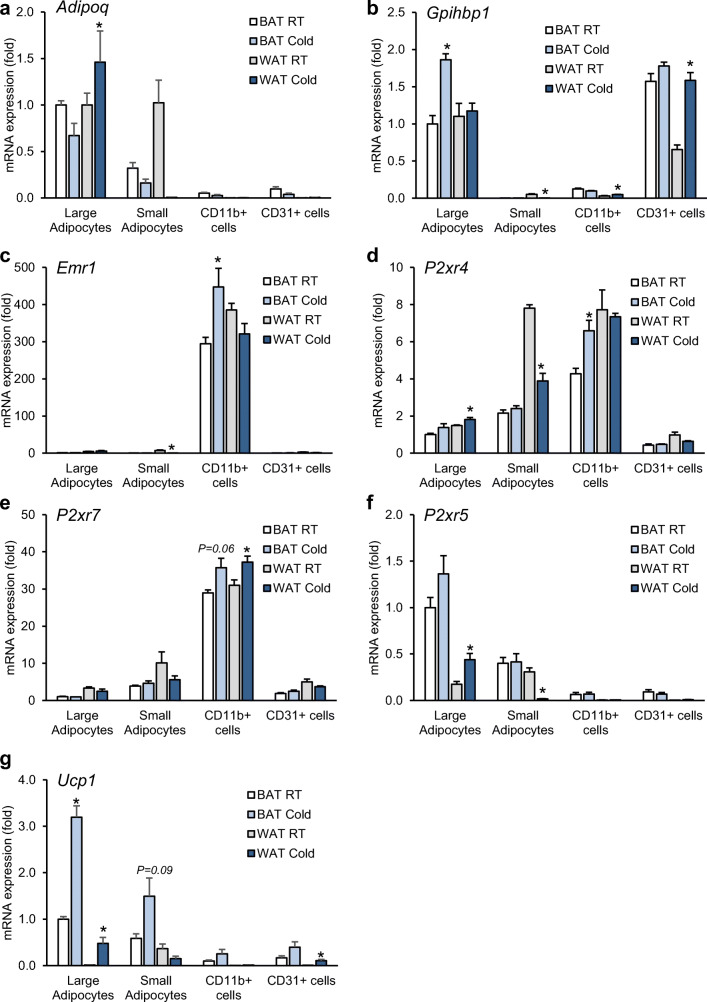
Gene expression analysis in cell fractions from adipose tissue (**a**–**g**). Male C57BL6/J WT mice were housed for 1 day at RT (22 °C) or cold (4 °C) condition. iBAT and subWAT tissues were harvested from each group, and different cell types were isolated by MACS®. Purity of cellular fractions was verified by gene expression of cell-specific markers for adipocytes (*Adipoq*), endothelial cells (*Gpihbp1*), and tissue resident macrophages (*Emr1*). The expression of *P2rx4*, *P2rx5*, *P2rx7*, and *Ucp1* was determined in all isolated cell fractions. *n* = 4. Statistical analysis was done between cells of RT and cold group isolated from each tissue. Data are presented as mean ± SEM, *P < 0.05

Cold exposure leads to a catabolic condition to promote energy combustion and heat production, which is associated with tissue remodeling of WAT and BAT [2, 28]. This prompted us to test the expression of purinergic receptors in adipose tissue upon cold exposure. To overcome the issue of cellular heterogeneity within adipose tissue, we determined the expression of receptors and enzymes regulating purinergic signaling in isolated cell types of WAT and BAT from mice under control (22 °C) and 1-day cold-activated (6 °C) conditions. We confirmed the purity of cellular fractions based on the expression of specific markers for adipocytes (*Adipoq*), endothelial cells (*Gpihbp1*) and tissue-resident macrophages (*Emr1*) (Fig. 2a–c). The expression of *P2rx4* was detected in both macrophages and adipocytes (Fig. 2d), while *P2xr7* was much higher in macrophages than other cell types (Fig. 2e). On the other hand, *P2xr5* was only present in brown adipocytes and a tendency of higher expression in response to cold exposure was observed (Fig. 2f), which is in accordance with the expression of *Ucp1* (Fig. 2g). Taken together, these data support the notion that purinergic P2X receptors could play a role in WAT and BAT remodeling in response to dietary or cold stress.

### Effect of P2X7 deficiency on cold-induced energy expenditure

**Fig. 3:**
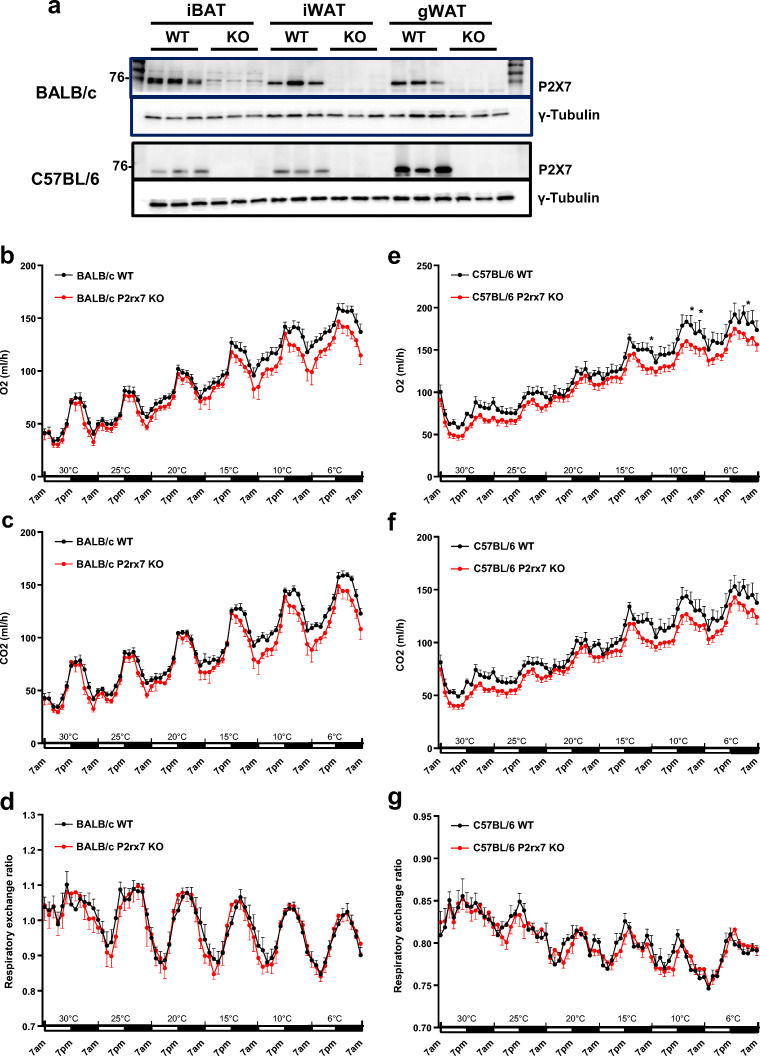
Comparative analyses of energy expenditure in WT and *P2rx7* KO mice. **a** Ablation of P2X7 protein in adipose tissues (iBAT, interscapular BAT; iWAT, inguinal WAT; gWAT, gonadal WAT) of male WT and *P2rx7* KO mice on the BALB/c and C57BL/6 backgrounds was confirmed by Western blotting. **b**–**g** Systematic energy expenditure in obese WT and *P2rx7* KO mice on the BALB/c and C57BL/6 backgrounds were determined by indirect calorimetry. Male WT and *P2rx7* KO mice were kept in metabolic monitoring cages for 1 week with a gradual decrease in housing temperature from 30 to 6 °C. O_2_ consumption, CO_2_ production, and respiratory exchange ratio (RER) were determined in **b**–**d** BALB/c and **e**–**g** C57BL/6 mice. For all experiments, *n* = 6 mice per group. Data are presented as mean ± SEM, *P < 0.05

Two knockout mouse models have been generated to study P2rx7 function. Pfizer established *P2rx7*-deficient mice by inserting a neomycin cassette into exon 13, replacing part of the C-terminus of the receptor, whereas GlaxoSmithKline generated them by inserting a LacZ transgene and neomycin cassette into exon 1 [29, 30]. In the current study, we performed metabolic studies in the mouse model generated by Pfizer, which expresses the P2X7 ΔC variant with reduced function [31]. We compared these knockout mice backcrossed onto the BALB/c and C57BL/6 backgrounds as BALB/c mice express the functional P451 allele while C57BL/6 mice express the low activity variant 451L. Protein expression analyses of interscapular BAT and inguinal and gonadal WAT from wild-type and *P2xr7* KO male mice that were fed a high-fat diet for 16 weeks showed the presence of P2X7 in all adipose tissue depots in both strains (Fig. 3a). As expected, the knockout was confirmed on the BALB/c as well as on the C57BL/6 background.

To determine whether P2X7 is involved in the regulation of adaptive thermogenesis in vivo, we measured oxygen consumption and carbon dioxide production in a temperature-controlled indirect calorimetry system. After adaptation to thermoneutral conditions (30 °C), we measured energy expenditure during the gradual decrease of the ambient temperature (5–6 °C each day) to 6 °C (Fig. 3b–g). Each drop in the temperature resulted in increased O_2_ consumption (Fig. 3b, e) and CO_2_ production (Fig. 3c, f). However, no significant differences were observed between wild-type and *P2rx7* KO male mice on the BALB/c background regarding O_2_ consumption, CO_2_ consumption, and respiratory exchange ratio (RER; Fig. 3b–d). Although upon gradual cold exposure, *P2rx7* KO male mice on the C57BL/6J background exhibited slightly reduced O_2_ consumption at some time points at low ambient temperature, CO_2_ consumption and RER were not significantly altered (Fig. 3e–g). Also, other metabolic parameters including food and water intake, and locomotor activity showed no significant differences between wild-type and *P2rx7* KO mice in both mouse strains studied (Supplementary Fig. 1a–f). Taken together, these results indicate a minor role for P2X7 in regulating energy expenditure during cold adaptation.

### Effect of P2X7 deficiency on adiposity

**Fig. 4:**
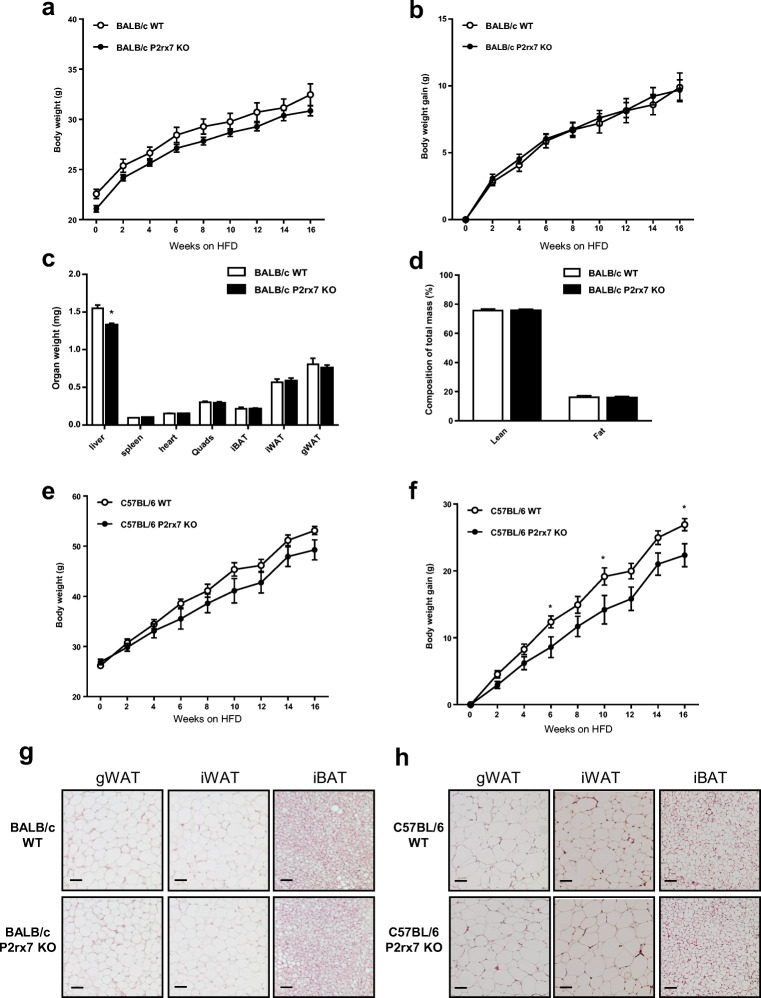
Diet-induced obesity studies in WT and *P2rx7* KO mice on the BALB/c and C57BL/6 backgrounds. Male WT and *P2rx7* KO mice were kept on a HFD for 16 weeks. **a** Body weight, **b** body weight gain, **c** organ weights, and **d** body fat composition was determined in *P2rx7* KO mice on BALB/c background. **e** Body weight and **f** body weight gain was determined in *P2rx7* KO mice on C57BL/6 background. *n* = 5–10 mice per group. **g**, **h** Representative microphotographs of gWAT, iWAT, and iBAT after *hematoxylin* and eosin (H&E) staining in *P2rx7* KO vs. WT mice. Data are presented as mean ± SEM, *P < 0.05

- In order to determine the effect of P2X7 deficiency on adiposity, we subjected *P2rx7* KO and wild-type control male mice on the BALB/c and C57BL/6 backgrounds to a HFD feeding regimen. After 16 weeks of HFD feeding, *P2rx7* KO on the BALB/c background showed no significant difference in body weight and body weight gain compared with WT controls (Fig. 4a, b). Of note, the weight of the liver was significantly reduced by deficiency of *P2rx7* on BALB/c (Fig. 4c) but not on C57BL/6 background (see also Fig. 7a, b). To note, there was no significant change in fat mass or lean mass upon HFD feeding in *P2rx7* KO mice on BALB/c background (Fig. 4d). *P2rx7* KO mice on the C57BL/6 background showed a tendency to lower body weight (Fig. 4e) and exhibited significantly reduced body weight gain at weeks 6, 10, and 16 of HFD feeding compared with WT controls (Fig. 4f). Histological analyses at the end of the experiment (HFD week 16) did not reveal any significant differences in adipocyte structure, size or number in gonadal WAT, inguinal WAT, or interscapular BAT (Fig. 4g, h) between *P2rx7* KO and WT mice. These results suggest that P2X7 deficiency does not have a major impact on adiposity during a HFD regimen.

### Effect of P2X7 deficiency on inflammation in adipose tissue

**Fig. 5:**
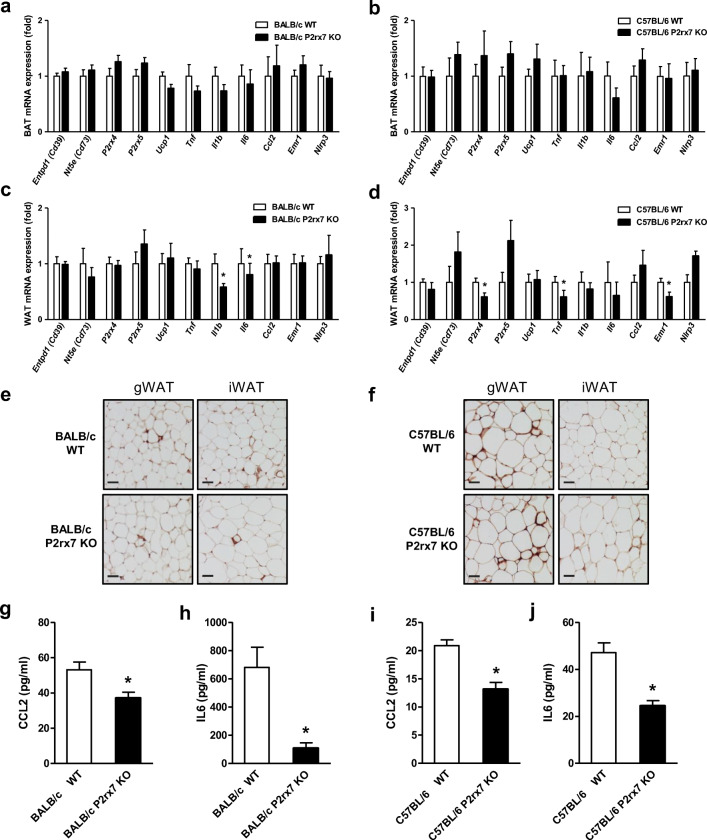
Inflammatory status in BAT and WAT of obese WT and *P2rx7* KO mice on the BALB/c and C57BL/6 backgrounds. Male WT and *P2rx7* KO mice were fed HFD for 16 weeks, and the gene expression of inflammatory markers was determined in **a**, **b** BAT and **c**, **d** gWAT by qPCR. **e**, **f** Representative microphotographs of anti-MAC-2 immune staining in gonadal (gWAT) and inguinal (iWAT) adipose tissues of WT and *P2rx7* KO mice on HFD. Plasma **g**, **i** CCL2 and **h**, **j** IL6 levels were determined in HFD fed WT and *P2rx7* KO mice on the **g**, **h** BALB/c and **i**, **j** C57BL/6 backgrounds by ELISA. *n* = 5–10 mice per group. Data are presented as mean ± SEM, *P < 0.05

It has been shown that P2X7 is involved in the activation of the inflammasome and stimulates the release of pro-inflammatory cytokines such as *IL1β* [32–34]. To determine the inflammatory state in adipose tissue of *P2xr7* KO male mice on both BALB/c and C57BL/6 backgrounds in response to HFD feeding, we performed gene expression analysis of genes encoding key players of purinergic signaling (*Entpd1*, *Nt5e*, *P2rx4*, *P2xr5*) and the inflammatory response (*Tnf*, *Il1b*, *Il6*, *Ccl2*) in BAT (Fig. 5a, b) and WAT (Fig. 5c, d). The results did not show any significant differences in the expression levels of these genes in BAT (Fig. 5a, b). In gonadal WAT of both BALB/c and C57BL/6 mice, we found a slight reduction in the expression of genes encoding pro-inflammatory cytokines and markers of infiltrating immune cells in *P2rx7* KO vs. WT mice (Fig. 5c, d). Consistently, we observed a modest reduction in macrophage infiltration in gonadal and inguinal WAT of *P2rx7* KO compared with wild-type control mice by immunohistochemistry using antibodies against the macrophage marker Galectin-3 (Mac-2) (Fig. 5e, f). Moreover, we found a robust decrease in circulating CCL2 and IL6 plasma levels after 16 weeks on HFD in *P2rx7* KO vs. WT mice in both strains (Fig. 5g–j). IL1-beta and TNF-alpha could not be detected by the method used. These findings indicate a decreased systemic inflammation in response to HFD feeding in *P2rx7* KO vs. WT mice.

**Fig. 6:**
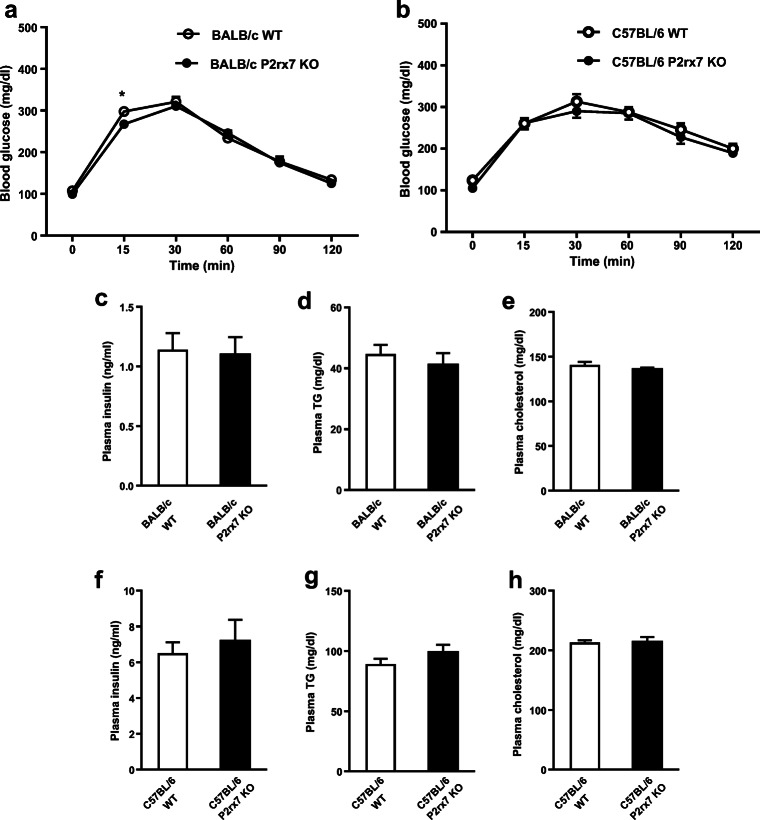
Glucose homeostasis in obese WT and *P2rx7* KO mice. **a**, **b** Glucose tolerance test in HFD fed male WT and *P2rx7* KO mice on the **a** BALB/c and **b** C57BL/6 backgrounds. Plasma **c**, **f** insulin, **d**, **g** triglyceride, and **e**, **h** cholesterol levels were measured using commercial kits. *n* = 5–10 mice per group. Data are presented as mean ± SEM, *P < 0.05

Next, we determined the impact of P2X7 deficiency on glucose homeostasis and insulin sensitivity in HFD-induced obesity. Glucose homeostasis, as determined by the oral glucose tolerance test (Fig. 6a, b) and by plasma levels of insulin (Fig. 6c, f), triglycerides (Fig. 6d, g), and cholesterol (Fig. 6e, h), was similar between *P2xr7* KO and WT male mice on both BALB/c and C57BL/6 backgrounds. Overall, these results suggest that P2X7 has a moderate effect on the inflammatory milieu in WAT but has only little if any influence on whole body glucose homeostasis.

### Effect of P2X7 deficiency on hepatic inflammation

**Fig. 7:**
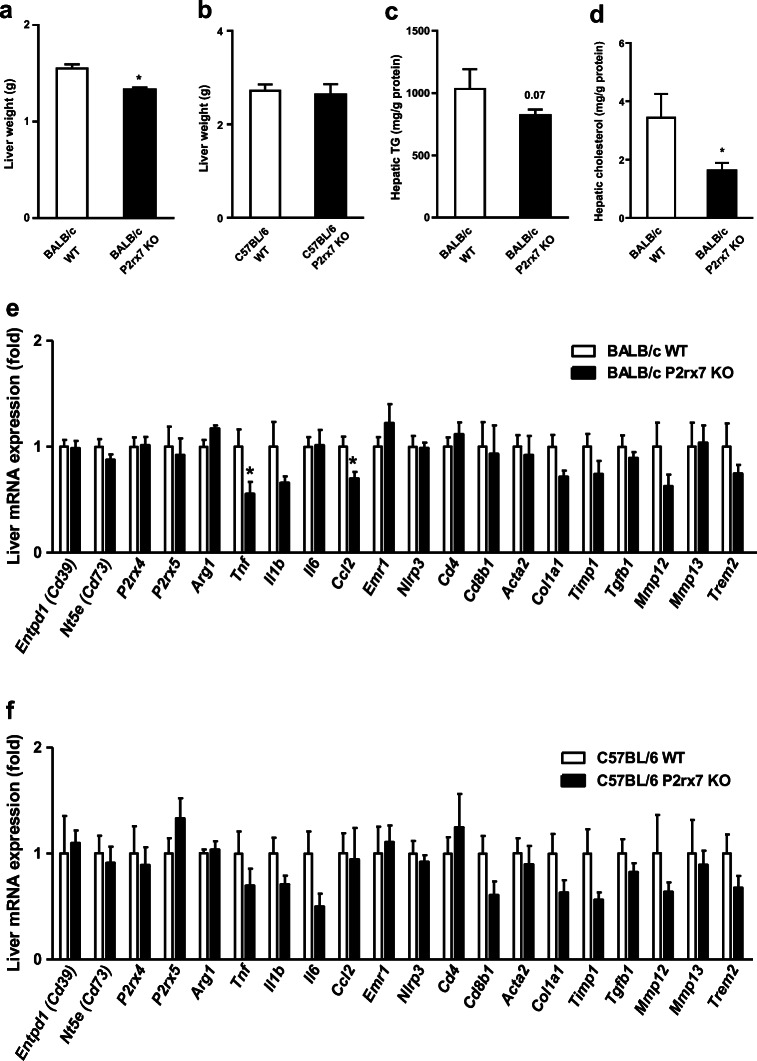
Effect on liver metabolic parameters in obese WT and *P2rx7* KO mice. Male WT and *P2rx7* KO mice were kept on a HFD for 16 weeks. **a**, **b** Liver weight was determined in *P2rx7* KO mice compared with WT controls on the **a** BALB/c and **b** C57BL/6 backgrounds. Liver **c** triglyceride (TG) and **d** cholesterol levels were determined and normalized to protein concentration. **e**, **f** Gene expression of inflammatory markers in the liver of WT and *P2rx7* KO mice on the **e** BALB/c and **f** C57BL/6 background. *n* = 5–10 mice per group. Data are presented as mean ± SEM, *P < 0.05

The results presented so far indicate that P2X7 plays only a minor role in metabolic inflammation in adipose tissue. On the other hand, we observed lower IL6 and CCL2 plasma levels (Fig. 5g–j) and in parallel lower liver tissue weight as well as hepatic triglyceride and cholesterol levels (Fig. 7a–d) in *P2rx7* KO male mice on the BALB/c background. This prompted us to perform gene expression analysis to determine the inflammatory state in the liver of *P2rx7* KO male mice after 16 weeks of HFD feeding. Compared with wild-type mice, we found reduced gene expression of *Tnf* and *Ccl2* in liver of *P2rx7* KO BALB/c mice (Fig. 7e). On the C57BL/6 background, there was a similar trend towards reduced expression, but this did not reach statistical significance (Fig. 7f). These results indicate that systemic inflammation in response to HFD-feeding is modulated more by hepatic P2X7 than by adipose P2X7.

## Discussion

The P2X7 ion channel is highly expressed in immune cells and is also found in other cell types such as epithelia, endothelia, osteoblasts, and fibroblasts [35]. Upon pro-inflammatory stimulation and activation, P2X7 coordinates cytokine release, changes in plasma membrane lipid distribution, and cell death processes [14, 17, 36]. In humans, visceral and subcutaneous adipose tissues express functional P2X7 that can be involved in secretion of inflammatory cytokines such as IL6, TNFα, and PAI1 [37]. In obese individuals, increased expression of P2X7 correlates with NLRP3 inflammasome activation and secretion of IL1β [38]. This was correlated with body mass index and metabolic syndrome score. It remains unclear which cell type (adipocytes or infiltrating immune cells) mediates this process. On the other hand, studies performed using mouse model do not show the involvement of P2X7 in obesity-associated inflammasome activation [39]. Sun et al. showed that *P2rx7* KO mice on the C57BL/6 background were not protected from diet-induced obesity and associated adipose inflammation. In another study using 9-month-old male *P2rx7* KO mice, P2X7 was suggested to have a role in adipogenesis and lipid metabolism [40]. However, these authors also did not detect any significant effects on metabolic parameters in females as well as 6-month or 12-month-old male *P2rx7* KO mice, which is in line with the data of the current study. Thus, the role of P2X7 in adipocyte metabolism and intercellular communication under pathophysiological condition remains unclear.

In adipose tissue, extracellular ATP is mainly released from presynaptic neural cells of sympathetic nerves. CD39 is a rate limiting ectoenzyme that hydrolyses ATP/ADP to AMP, while CD73 degrades AMP to adenosine. Thus, the enzymatic activities of CD39 and CD73 play crucial roles in determining duration and magnitude of purinergic signaling [41]. Recently, it was shown that ATP acting via P2X7 promotes a Th17 polarizing microenvironment in WAT [42]. In vivo studies have shown a role for CD39 in insulin signaling and liver metabolism, whereas CD73 plays a role in intracellular lipid metabolism in adipose tissue and muscle [43–45]. We do not have insight into the relevance of this extracellular purinergic signaling in adipose tissue for metabolic cross-talk between infiltrating and tissue-resident immune cells and paracrine signaling between immune cells and adipocytes. Here we show that both nutritional stress and genetic model of obesity result in increased expression of P2X7 which is associated with increased macrophage infiltration within adipose tissue and also increased expression of CD39 and CD73. Adipose tissue shows high heterogeneity, wherein at least half of the cells are non-adipocytes and is composed of several cell types namely adipocytes, fibroblasts, immune cells, and endothelial cells [46, 47]. Multiple cell types add to the complexity of the cellular interaction in adipose tissue and the relative expression and distribution of P2X7 within different cell types of adipose tissues have not been fully elucidated. Through cell separation studies in adipose tissue, we show the robust expression of P2X7 in macrophages compared with other cell types. These results are critical towards understanding the cell type–specific contribution of *P2rx7* during the progression of obesity and macrophage infiltration.

We sought to ascertain the role of P2X7 in the diet-induced obese mouse model. Previous studies have investigated the role of P2X7 in disease states using *P2rx7*-deficient mice on the C57BL/6 background. However, the C57BL/6 mouse strain has a naturally occurring mutation that leads to replacement of the proline residue at position 451 by a leucine residue in the cytoplasmic tail of P2X7 [20]. The distribution of the allelic version of P451L SNP is different in BALB/c and C57BL/6 mice. BALB/c mice and C57BL/6 carry P451 and 451L alleles, respectively. P2X7 functional studies show that T cells from BALB/c mice have significantly higher sensitivity to ATP than those obtained from C57BL/6 mice [20]. Apart from in vitro studies, the P451L SNP has been implicated in disease development in two studies. First, an association between the P451L SNP and neuropathic pain in mice has been reported in genome-wide linkage analysis studies [23]. Second, a bone phenotype was found to be associated with P451L SNP in C57BL/6 mice [24]. Based on these studies, it has been suggested that the P451L SNP may underestimate purinergic receptor function in *P2rx7* KO mice on the C57BL/6 background compared with BALB/c mice [20, 48]. In order to address the impact of strain differences on metabolic phenotype, we compared the pathophysiological events in *P2rx7* KO mice on both, C57BL/6 and BALB/c backgrounds. Notably, P2X7 deficiency in both the strains did not influence glucose intolerance or insulin resistance associated with diet-induced obesity. Moreover, neither body weight nor adiposity was altered by *P2rx7* deficiency. Also, plasma parameters such as glucose, insulin, triglycerides, and cholesterol levels were unchanged, indicating that even the full functional P2X7 receptor of BALB/c mice does not play a major role in adipose metabolic inflammation.

There have been other recent studies using *P2rx7* KO mice on the C57BL/6 background, and as is a typical of metabolic phenotyping studies, our findings show some striking similarities as well as some important differences. Consistent with our work, Sun et al. did not observe any significant change in body weight, insulin sensitivity, or inflammatory state upon 12-week HFD feeding in *P2rx7* KO mice on the C57BL/6 background [39]. On the other hand, Beaucage et al. reported that 9-month-old male *P2rx7* KO mice on the C57BL/6 background exhibited increased body weight, epididymal fat pad weight, and ectopic lipid accumulation in kidney, pancreas, and extraorbital lacrimal gland [40]. However, no significant metabolic differences were observed in female, and younger (< 6 months) or older (> 12 months) mice [40]. Taken together, these data suggest that *P2rx7* may play only a minor role if any in insulin resistance caused by overnutrition in mouse models.

Addressing the effect of P2X7 on BAT is complex and may be different between rodents and humans [49]. P2X7 has been considered important for cellular energy homeostasis, whole body energy metabolism, and fatty acid oxidation [50], although our data question this notion. Giacovazzo et al. determined energy expenditure, O_2_ consumption, and RER in a narrow window of 24-h acclimatization followed by 24-h measurement using 20-month-old female mice. We found on both strains that P2X7 deficiency did not result in significant alterations in energy expenditure or fuel utilization. Also, the gene expression of *Ucp1* and other thermogenic markers was not significantly altered in BAT when *P2rx7* was inactivated. Our in vivo studies showed increased *P2rx5* mRNA level in brown adipocytes compared with other cell types in BAT that was induced upon cold exposure (Fig. 2f). These results are consistent with previous study identifying *P2rx5* as a novel cell surface marker for brown adipocytes in mice and humans [51–53]. Despite no change in *P2rx5* mRNA levels in BAT, *P2rx7* KO mice on HFD showed increased P2X5 protein levels compared with WT controls indicating a potential compensatory mechanism between *P2rx5* and *P2rx7* (Supplementary Fig. 2a). Interestingly, *P2rx4* mRNA expression is reduced in WAT of *P2rx7*-deficient mice (Fig. 5d), which may points towards a mutual regulation of these P2x receptors within adipose tissues under conditions of high caloric intake. Taken together, our data suggest that P2X7 exerts only minor effects on the thermogenic function of BAT. One possible interpretation of the finding that *P2rx7* is dispensable for the development of diet-induced obesity and insulin resistance is that other members of the P2X receptor family alone or in combination with other members may play a more important role in these processes. Probably, also aging-related metabolic adaptation may play a role in manifesting metabolic dysfunctional processes. In such a scenario, identifying interacting partners through IP and Co-IP experiments would help shed light on these pathways.

One interesting result from our study was that *P2rx7* KO mice showed reduced liver weight, liver triglycerides, and cholesterol levels upon HFD feeding. Furthermore, the reduced expression of pro-inflammatory genes and reduced levels of plasma cytokines CCL2 and IL6 in *P2rx7* KO vs. WT mice may indicate a role for P2X7 in liver upon nutritional inflammatory stimulus. Surprisingly, a recent study reported dyslipidemia and hepatic steatosis along with increased weight gain in *P2rx7* KO mice [54]. In this study, lipogenic pathways were shown to be affected, indicating that P2X7 deficiency may deregulate liver function under severe hepatic pathological conditions.

In conclusion, even in BALB/c mice, which in contrast to C57BL/6 mice express the fully functional receptor, P2X7 deficiency does not have a significant effect on the function of adipose tissues under conditions of dietary or cold stress. The emerging picture suggests that a possible role of P2X7 is not mediated by HFD-induced adipose tissue hypertrophy and inflammation but may rather be modulated by inflammation in the liver. It is also possible that the murine P451L SNP exerts critical cell type–specific effects that contribute to the diversity of receptor-mediated responses in studies addressing the role of P2X7 in health and disease.

## Electronic supplementary material

Supplementary Figure 1. Comparative analyses of energy expenditure in WT and *P2rx7* KO mice. (**a,d**) Food intake, (**b,e**) water intake and (**c,f**) locomotor activity was measured in male (**a-c**) BALB/c and (**d-f**) C57BL/6 mice. *n*=6 mice per group. Data are presented as mean ± SEM. (PPTX 281 kb)

Supplementary Figure 2. Expression level of P2X5 by Western blotting in male WT and *P2rx7* KO mice on HFD (**a**). (PPTX 281 kb)
