# Supplementary figures and images for: The P2X7 ion channel is dispensable for energy and metabolic homeostasis of white and brown adipose tissues
Source: Purinergic Signal. 2020 Oct 6;16(4):529–42. doi: 10.1007/s11302-020-09738-7 (PMC7855144; doi:10.1007/s11302-020-09738-7)

## Slide 1
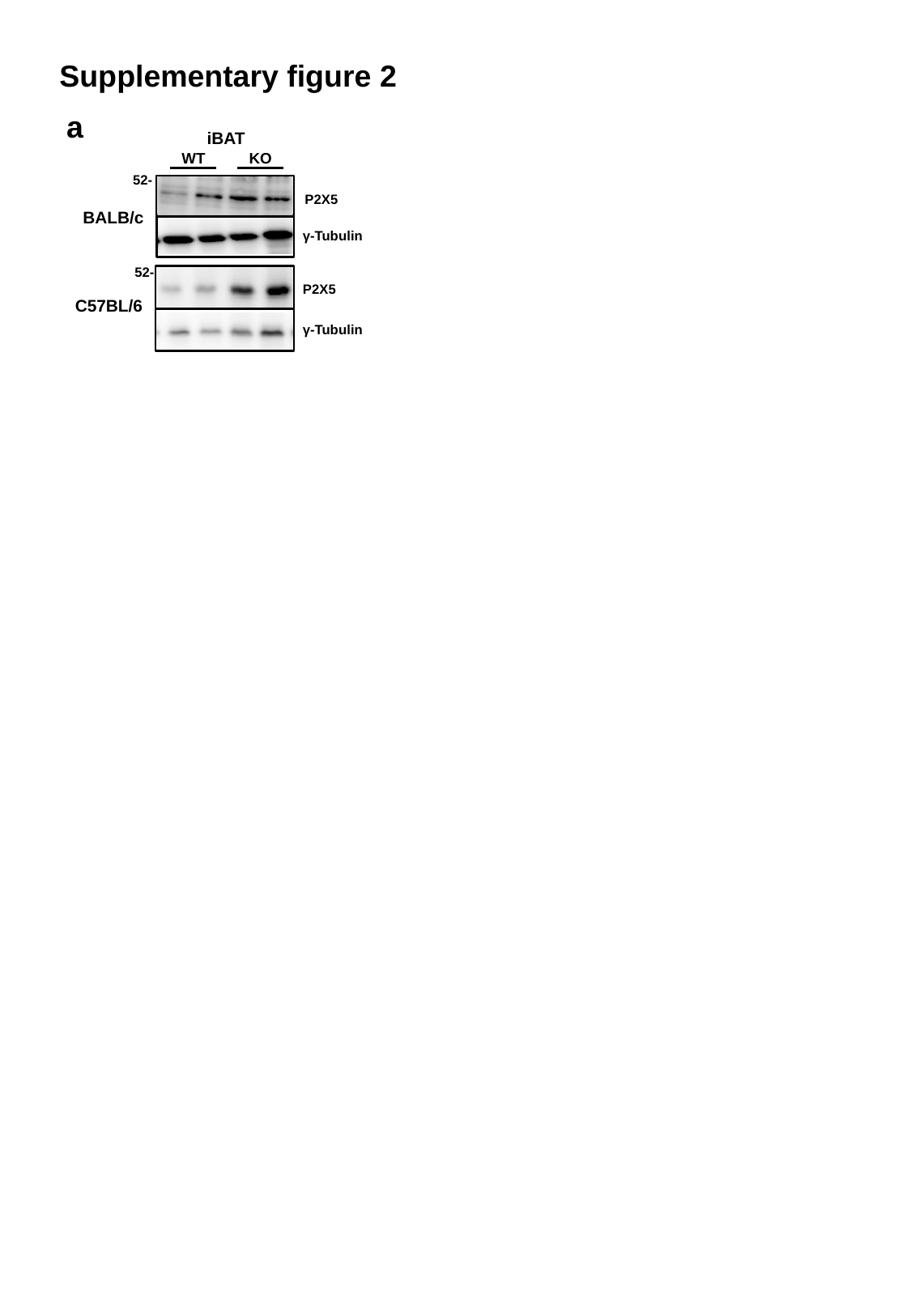

Supplementary figure 2
a
iBAT
WT
KO
52-
P2X5
BALB/c
γ-Tubulin
52-
P2X5
C57BL/6
γ-Tubulin

Supplement: Supplementary file 2 — Expression level of P2X5 by Western blotting in male WT and P2rx7 KO mice on HFD (a). (PPTX 281 kb) [file 11302_2020_9738_MOESM2_ESM.pptx]
